# Semantic differential analysis of effects of indoor soundscapes on learning efficiency during online home-based classes

**DOI:** 10.1371/journal.pone.0306812

**Published:** 2024-08-15

**Authors:** Dahu Lin, Tingjun Li, Haijuan Liang

**Affiliations:** School of Architecture and Art, Hebei University of Architecture, Zhangjiakou, Hebei, China; Shahid Beheshti University, ISLAMIC REPUBLIC OF IRAN

## Abstract

This investigation into the effects of indoor soundscapes on learning efficiency during home-based online classes amidst the COVID-19 pandemic leveraged a questionnaire survey to gather insights from participants across 32 provinces in China. The survey findings reveal a notable preference among respondents for sounds emanating from nature and culture, alongside an acceptance of sounds inherent to lectures. A significant majority showed a preference for a tranquil soundscape or one enriched with natural and cultural elements, emphasizing that such an environment, coupled with the ability for active communication, is conducive to enhancing learning efficiency. Through semantic differential analysis, the study identified four pivotal factors that influence subjective evaluations of indoor soundscapes: the nature of online classes, relaxation, physical attributes of the soundscape, and aspects related to personal study. Additionally, the analysis delved into gender and regional differences in soundscape perceptions and their impact on learning. A key finding is that complex soundscapes negatively affect the learning process, with 45.7% of respondents reporting a perceived decrease in learning efficiency attributable to the indoor soundscape experienced during home-based online classes. Consequently, this study suggests that optimizing learning efficiency requires creating simpler, lighter, quieter, and more relaxing soundscapes. These insights hold both theoretical and practical value, offering a foundational basis for further research into indoor soundscapes and informing the development and management of online classes. The findings underscore the importance of considering the auditory environment as a critical component of effective online education, highlighting the need for strategies that mitigate auditory distractions and foster an acoustically conducive learning space.

## 1. Introduction

In many countries, online classes became the predominant mode of learning during the COVID-19 pandemic, facilitated by developments in network technology. However, the soundscapes during online classes can be complex and diverse. Therefore, investigating the impact of the soundscape on learning within online environments is essential, highlighting its significance as a pivotal factor. Schafer [1] conducted pioneering research into soundscapes, laying the groundwork for numerous subsequent studies from diverse perspectives and various environments. The International Organization for Standardization defines a soundscape as the acoustic environment in a specific location as experienced by individuals, groups, or communities [2]. While there has been extensive research on soundscapes across different environments, from urban public spaces [3] to forests, villages, campuses [4–6], and even studies comparing soundscapes across different cultural contexts [7], most of these studies are focused on leisure environments. Investigations into indoor soundscapes within home online learning settings and their impact on learning efficiency remain relatively scarce. In the realm of indoor soundscapes, Volkan et al. [8] studied the indoor soundscapes in open learning areas and extracted three key factors: sensation, activity/communication, and functionality. Simone et al. [9] investigated residential indoor soundscapes and extracted three factors: comfort, content, and familiarity. Zhen et al. [10] utilized semantic differential analysis to investigate the soundscape in a school library and proposed an optimal design strategy.

Although research on educational spaces’ indoor soundscapes is limited, existing studies demonstrate a significant impact on learning environments. For instance, the indoor soundscape study by Dokmeci Yorukoglu and Kang in university libraries [11], the conceptual framework developed by Cankaya and Yilmazer for high school environments [12], and Acun and Yilmazer’s research on open learning spaces [8, 13] not only highlight the soundscape’s potential effect on learning efficiency but also offer theoretical and methodological guidance for this research.

Confronting the new reality of home-based online learning during the COVID-19 pandemic, the study of indoor soundscapes’ impact on learning efficiency becomes critically urgent. To address this, our study aims to explore the following research questions:

1. Do the physical properties, personal and social attributes, and educational attributes of indoor soundscapes have an impact on students’ learning efficiency during online home learning?
2. Which key factors of indoor soundscapes will affect the efficiency during online home-based classes? And how does this differ from semantic differential analysis of other soundscapes?
3. How to effectively improve learning efficiency during online home-based classes?

By addressing these research questions, this study employs the semantic differential method, complemented by surveys and field tests with students from 32 provinces in China, to thoroughly understand indoor soundscapes during home-based online classes and their effect on learning efficiency. This study aims to provide an empirical foundation and specific strategies for optimizing online learning environments, particularly by improving the acoustic conditions in home learning settings, thereby boosting learning efficiency.

## 2. Methodology

This study employs the semantic differential method, leveraging surveys and field tests to investigate the indoor soundscape during home-based online classes. Utilizing statistical analysis tools, it analyzes the impact of the indoor soundscape on learning efficiency within this context.

### 2.1. Subjective evaluation of the soundscape

Subjective evaluations of soundscapes have traditionally relied on methods such as soundscape walks [14], semantic differential analysis [15–17], interviews [18], experimental approaches [19], and combined methods incorporating both physical measurements and subjective evaluations [20]. Notably, semantic differential analysis, introduced by Osgood et al. [21], identifies the emotional connotations of words. This technique has been widely adopted across various fields, playing a crucial role in the quantitative assessment of soundscapes.

In this study, the semantic differential method is utilized. Participants are prompted to rate sounds using a five-point bipolar scale, consisting of adjective pairs. The selection of these pairs presents two primary challenges: ensuring relevance to the community’s cultural, sociological, and linguistic contexts, and accurately describing the sound environment. The adjective pairs chosen for this study are derived from established soundscape literature.

Considering the typical scenario of students attending live lectures via online conferencing from home, alongside prior soundscape research [22–31], this study focuses on personal, physical, social, and educational attributes. Consequently, 30 antonym pairs were selected as the indicators for semantic differential analysis, including but not limited to boring/interesting, like/hate, and friendly/unfriendly. This analysis employs a seven-point scale, enabling respondents to evaluate and quantify the soundscape’s attributes based on their personal experiences during online classes. These evaluations facilitate the mapping of soundscapes within a semantic space. Additionally, to understand the actual semantic implications further, a questionnaire survey was conducted to examine the selected words’ specific meanings.

Twenty indicators were selected by analyzing and screening the results of the survey conducted among 36 undergraduate and postgraduate students with related majors. As shown in **Table 1**, the screened semantic indicators covered various aspects of the soundscape, including personal attributes (comfort/discomfort, mysterious/not mysterious, beautiful/not beautiful, like/dislike, and quiet/noisy), physical attributes (weak/strong, light/heavy, clear/mixed, ordered/disordered, pure/impure, single/diverse, and varied/simple), social attributes (relaxation/tension, friendly/unfriendly, and harmony/conflict), and educational attributes (concentration/distraction, interesting/boring, meaningful/meaningless, improved learning efficiency/decreased learning efficiency, and favorable for communication/unfavorable for communication).

**Table 1:** Soundscape evaluation attributes.

		Very	Fairly	Little	Neutral	Little	Fairly	Very	
Personal attributes	Comfort	3	2	1	0	–1	–2	–3	Discomfort
	Mysterious	3	2	1	0	–1	–2	–3	Not mysterious
	Beautiful	3	2	1	0	–1	–2	–3	Not beautiful
	Like	3	2	1	0	–1	–2	–3	Dislike
	Quiet	3	2	1	0	–1	–2	–3	Noisy
Physical attributes	Weak	3	2	1	0	–1	–2	–3	Strong
	Light	3	2	1	0	–1	–2	–3	Heavy
	Clear	3	2	1	0	–1	–2	–3	Mixed
	Ordered	3	2	1	0	–1	–2	–3	Disordered
	Pure	3	2	1	0	–1	–2	–3	Impure
	Single	3	2	1	0	–1	–2	–3	Diverse
	Varied	3	2	1	0	–1	–2	–3	Simple
Social attributes	Relaxation	3	2	1	0	–1	–2	–3	Tension
	Friendly	3	2	1	0	–1	–2	–3	Unfriendly
	Harmony	3	2	1	0	–1	–2	–3	Conflict
Educational attributes	Concentration	3	2	1	0	–1	–2	–3	Distraction
	Interesting	3	2	1	0	–1	–2	–3	Boring
	Meaningful	3	2	1	0	–1	–2	–3	Meaningless
	Improved learning efficiency	3	2	1	0	–1	–2	–3	Decreased learning efficiency.
	Favorable for communication	3	2	1	0	–1	–2	–3	Unfavorable for communication

### 2.2. Locations of participants

During the COVID-19 pandemic, students transitioned to receiving online classes from home, a significant departure from traditional in-person classes held in educational institutions. To investigate the impact of soundscapes on home-based online learning, questionnaires and home acoustic environment assessments were carried out. The residences of the respondents served as the experimental sites for this study. The delineation between North and South China was based on the climatic regions outlined in China’s building climate zoning map, with South China characterized as a mild region featuring hot summers and either cold or warm winters, and North China identified as a cold region experiencing severely cold winters.

**Fig 1:**
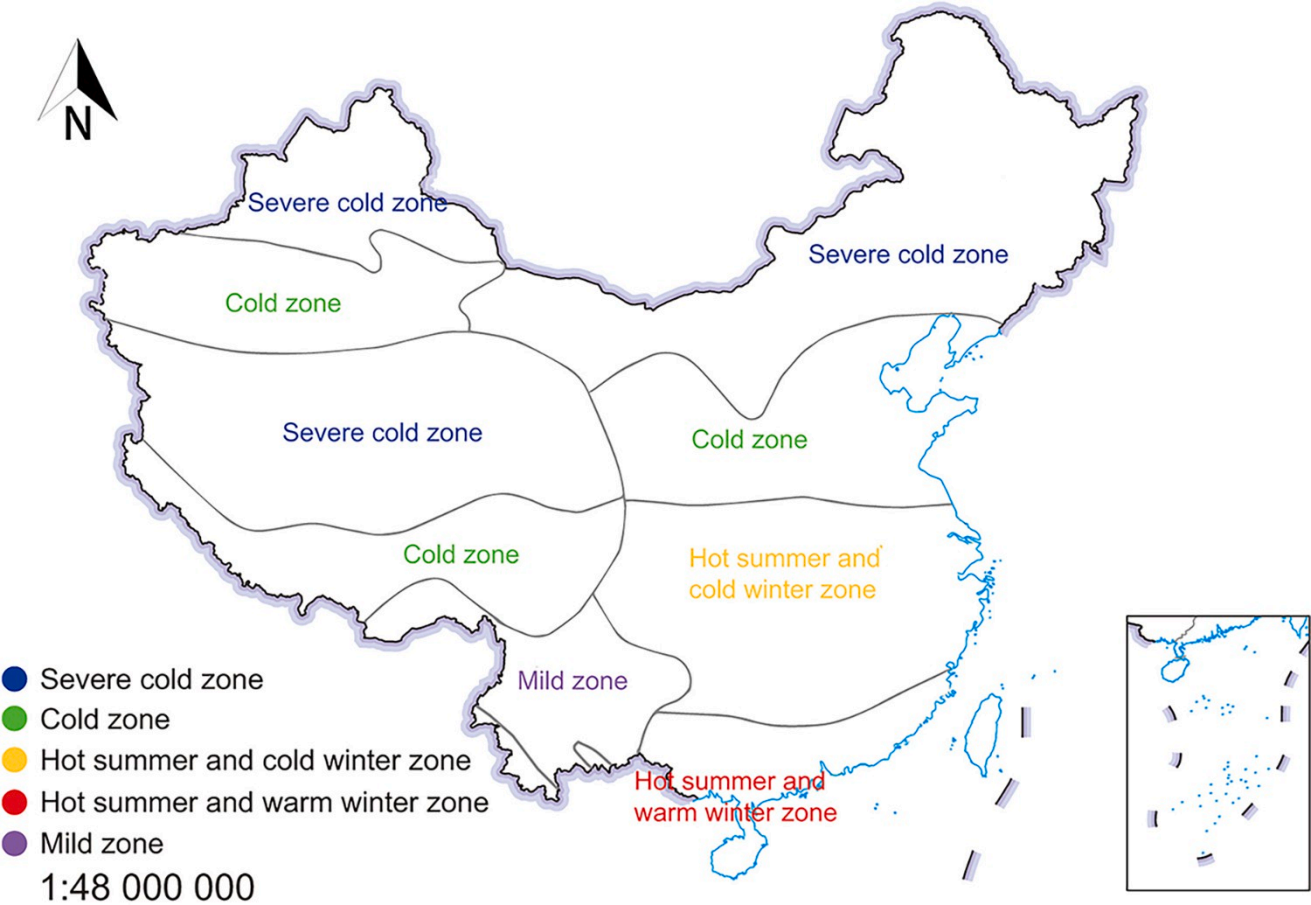
Distribution of climate zones in China.

As shown in **Fig 1**, participants hailed from 32 provinces in various climate regions of China, ensuring a broad and representative geographical distribution. Throughout the duration of the study, all respondents participated in online classes from within their homes. The findings from the survey regarding the indoor soundscapes are detailed in **Tables 2** and **3**. The elements constituting the indoor soundscape during online classes were categorized into keynote sounds, signals, and sound marks. Moreover, the sources of these soundscape components during home-based online learning were classified as geophonies (natural sounds), anthrophonies (human sounds), and biophonies (biological sounds), collectively forming a comprehensive framework of indoor soundscape elements pertinent to online education at home.

**Table 2:** Indoor soundscape composition system during the online class.

Category	Specific sound
Keynote sounds	Sound of wind, sound of rain, sound of cars driving or honking on the road, sound of parents or other relatives walking
Signals	Didi information prompt sound on WeChat/QQ and other apps, sound of children playing and crying, sound of TV at home
Marknotes	Teacher’s voice, music, conversations among parents and other relatives

**Table 3:** Indoor soundscape composition system during the online class according to the sound source.

Category	Specific sound
Geophonies	Sound of wind, sound of rain
Anthrophonies	Didi information prompt sound on WeChat/QQ and other apps, sound of TV at home, music, sound of cars driving or honking on the road
Biophonies	Teacher’s voice, the conversations among parents and other relatives, sound of children playing and crying, sound of parents or other relatives walking

### 2.3. Characteristics of study participants

The COVID-19 pandemic has underscored the utility of online courses as effective learning tools, albeit highlighting considerable variation in learner attributes. The demographic of students engaging in online learning encompassed a diverse group distinguished by gender, geographic location, and educational attainment. This study involved surveying 1,105 respondents across 32 provinces in North and South China, aiming to examine the nuances of their home-based learning experiences. Through a questionnaire survey and self-assessment, participants evaluated their acoustic environments at home, yielding 951 valid responses.

The survey data, summarized in **Table 4**, indicate a male participation rate of 53.7% and a female participation rate of 46.3%. Geographically, 389 respondents were from South China, while 562 hailed from North China, illustrating a wide geographic spread. The age distribution was predominantly between 21 and 25 years, accounting for over 76.9% of respondents. Additionally, the survey included 60 individuals aged 31 years or older, with the youngest four respondents being 17 years or younger.

**Table 4:** Demographic information for respondents.

	Classification	Number	Percentage
Gender	Male	511	53.7
	Female	440	46.3
Age	≤ 17 years	4	0.4
	18–20 years	95	10.0
	21–25 years	731	76.9
	26–30 years	61	6.4
	≥ 31 years	60	6.3
Educational level	High school degree and below	68	7.2
	College degree	41	4.3
	Bachelor’s degree	781	82.1
	Master’s degree	51	5.4
	PhD degree and above	10	1.1
Region	North	562	59.1
	South	389	40.9

Educational levels among the respondents varied: 68 were in high school or below, 41 were attending junior college, 781 were undergraduates, 51 were master’s students, and 10 were doctoral students or higher. These participants were healthy college students who transitioned to home-based online classes due to the pandemic and were reported to have normal vision, hearing, and psychological conditions. Having engaged with the online course format for a period prior to the study, respondents were well-placed to provide insightful feedback regarding their online learning experiences and acoustic environments at home.

### 2.4. Experimental design

#### 2.4.1. Questionnaire

The questionnaire was segmented into four sections: personal information, acoustic perception, semantic differential analysis scale, and field assessment of the acoustic environment. Personal information included the test date, gender, age, residence in South or North China, and level of education. Acoustic perception sought to gauge respondents’ feelings towards common indoor sound sources during online classes on a scale from 3 (very positive) to -3 (very negative), including an option for neutrality (0), and their conception of an ideal soundscape. The semantic differential analysis scale, featuring a seven-point range (-3 to 3), asked respondents to evaluate 20 semantic indicators encompassing aspects such as personal satisfaction, physical, social attributes, and learning efficiency. This method, effective for converting qualitative data into quantitative insights, is pivotal for soundscape analysis.

#### 2.4.2. Physical environment evaluation

In light of COVID-19, respondents engaged in home-based learning via online platforms. This study utilized questionnaires and field tests to assess indoor acoustic levels-averages, maxima, and minima-during online classes.

#### 2.4.3. Experimental procedure

The experiment took place in April and May 2020, amidst the pandemic, ensuring a robust respondent turnout as classes moved online. Participants, confined to home due to COVID-19, were randomly selected, amounting to 1,105 healthy online learners. To mitigate external influences on emotional responses, the use of electronic devices was restricted to educational purposes, and non-academic communication was minimized.

Participants were briefed on the experiment’s scope and urged to rest adequately before the class to ensure a typical learning state for accurate questionnaire completion. They filled out the initial part of the questionnaire prior to the class and logged sound levels throughout. During the class, they adopted a comfortable sitting position, engaging with the class content as usual. Post-class, a detailed questionnaire captured their auditory experiences, ideal soundscape preferences, and semantic assessments.

#### 2.4.4. Data analysis

The respondents entered the data online using an app named “Wenjuanxing”. All of the raw data were recorded and processed using Microsoft Excel. Origin 2021 and SPSS 26 were used for all data analyses.

## 3. Results

This section delineates the outcomes of our study on soundscapes and learning efficiency, incorporating evaluations and comparisons of sound levels and acoustic comfort, analysis of acoustic perception and sound preferences, the impact of demographic characteristics and the indoor soundscape on learning efficiency, and the identification of characteristic semantic factors related to the indoor soundscape during home-based online classes through semantic differential analysis.

### 3.1. Soundscape evaluation

#### 3.1.1. Subjective evaluations of sound level and acoustic comfort

**Fig 2:**
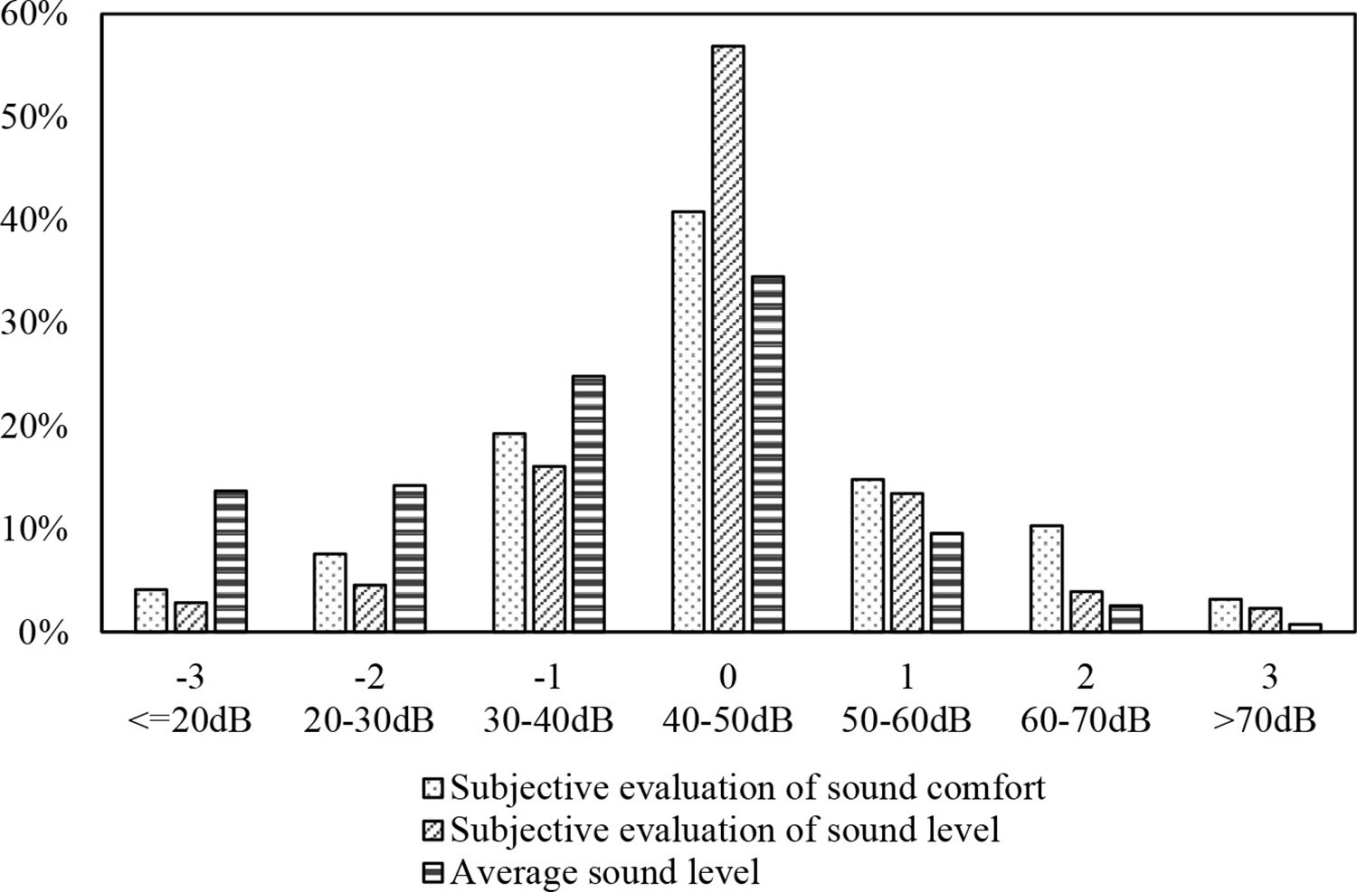
Distribution of sound pressure levels, sound comfort, and subjective loudness evaluation.

Subjective evaluations of loudness and acoustic comfort employed a 7-point scale, with loudness ratings spanning from -3 (very weak) to 3 (very strong). Similarly, comfort assessments ranged from -3 (extremely uncomfortable) to 3 (extremely comfortable). The average sound pressure level, as derived from actual data, was categorized into seven intervals: <20dB, 20–30dB, 30–40dB, 40–50dB, 50–60dB, 60–70dB, and >70dB. Table 5 presents a comparison of the standard deviation and mean for these metrics, alongside their normalized counterparts. **Fig 2** illustrates the distribution of these indicators, evidencing a general adherence to a normal distribution, albeit with minor discrepancies. The arithmetic means for both subjective assessments are closely aligned, as depicted in **Table 5** (mean subjective loudness rating: -0.055; mean sound comfort rating: -0.018). However, the standard deviation for sound comfort assessments exceeds that for loudness evaluations, suggesting a broader dispersion in comfort assessments, as illustrated in **Fig 2**. This disparity hints at a more nuanced range of subjective comfort evaluations compared to loudness evaluations. The average noise level was found to be 38.8dB, predominantly ranging between 30–50dB, with a minority exceeding 60dB.

**Table 5:** Statistical analysis of sound pressure levels, sound comfort, and subjective loudness evaluation.

	Raw statistics		Normalized statistics	
	standard deviation	average	standard deviation	average
Subjective evaluation of sound comfort	1.308	-0.018	0.130	0.143
Subjective evaluation of sound level	1.046	-0.055	0.196	0.143
Average sound level	13.1 dB	38.8 dB	0.120	0.142

A further examination of the correlation among subjective loudness evaluations, sound comfort assessments, and measured average sound levels disclosed no significant correlation (p < 0.01), marking a departure from findings in outdoor sound environment studies. This divergence may stem from the distinct sound requirements and expectations inherent to classroom settings as opposed to outdoor leisure contexts.

#### 3.1.2. Acoustic perception and acoustic preference during online classes at home

**Fig 3:**
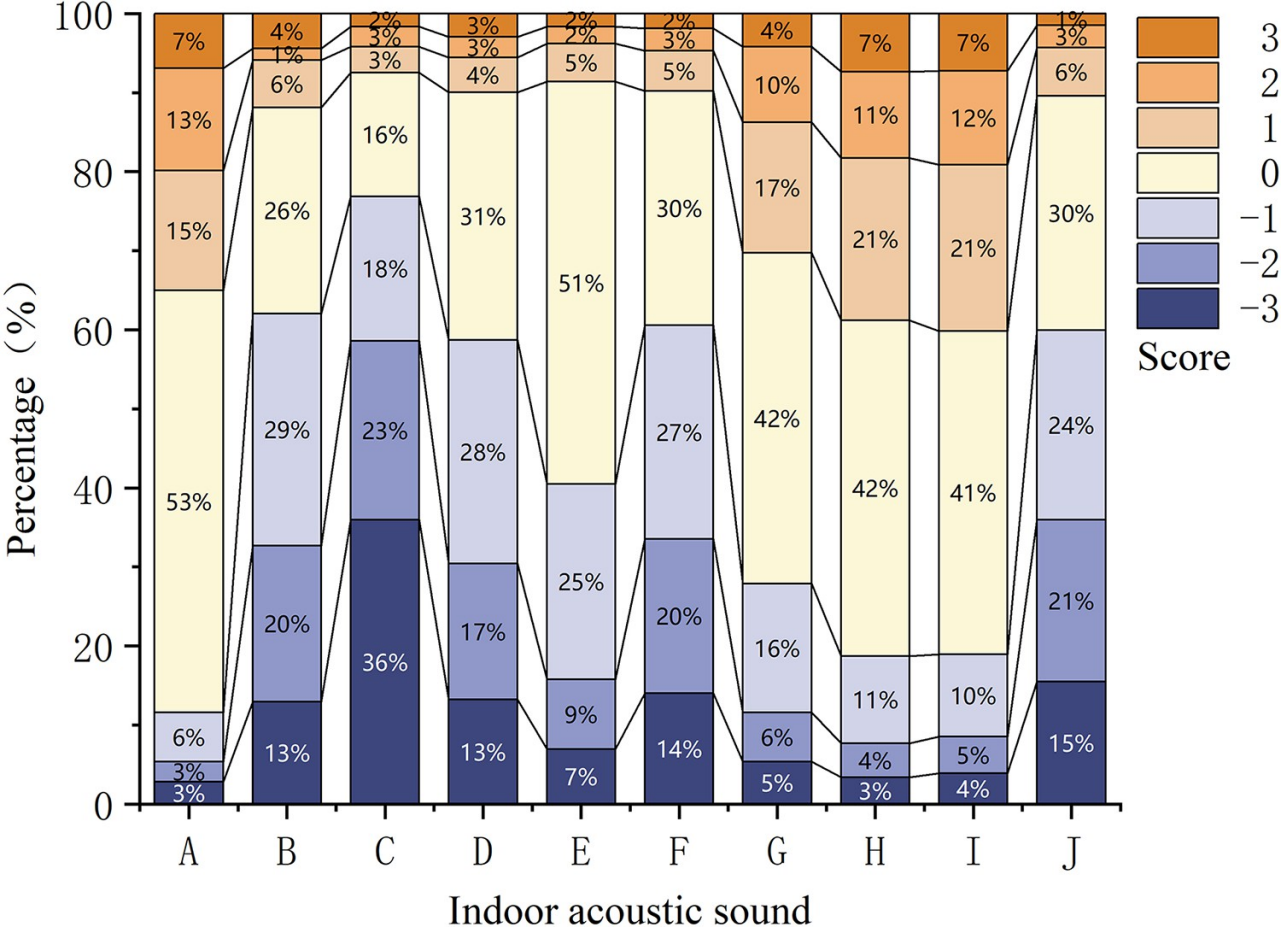
Votes for preferred sounds during online classes at home. A: Teacher’s voice; B: Sound of TV at home; C: Sound of children playing and crying; D Conversations among parents and other relatives; E: Sound of parents and other relatives walking; F: Sound of cars driving or honking on the road; G: Music; H: Sound of wind; I: Sound of rain; J: Notification sounds of messaging software.

**Fig 3** illustrates respondents’ reactions to the ambient sounds while participating in online classes from home. The teacher’s voice received the highest acceptance, although a minor segment of the participants voiced varying degrees of dissatisfaction with it. Generally, sounds associated with nature and culture were more favorably received. Specifically, the sounds of wind and rain were most preferred, followed closely by music. Conversely, the sounds of children playing and crying ranked as the least favored. Other less popular sounds included television noise within the home, conversations among parents and other family members, vehicular noises such as driving or honking, and notification sounds from messaging applications.

**Fig 4:**
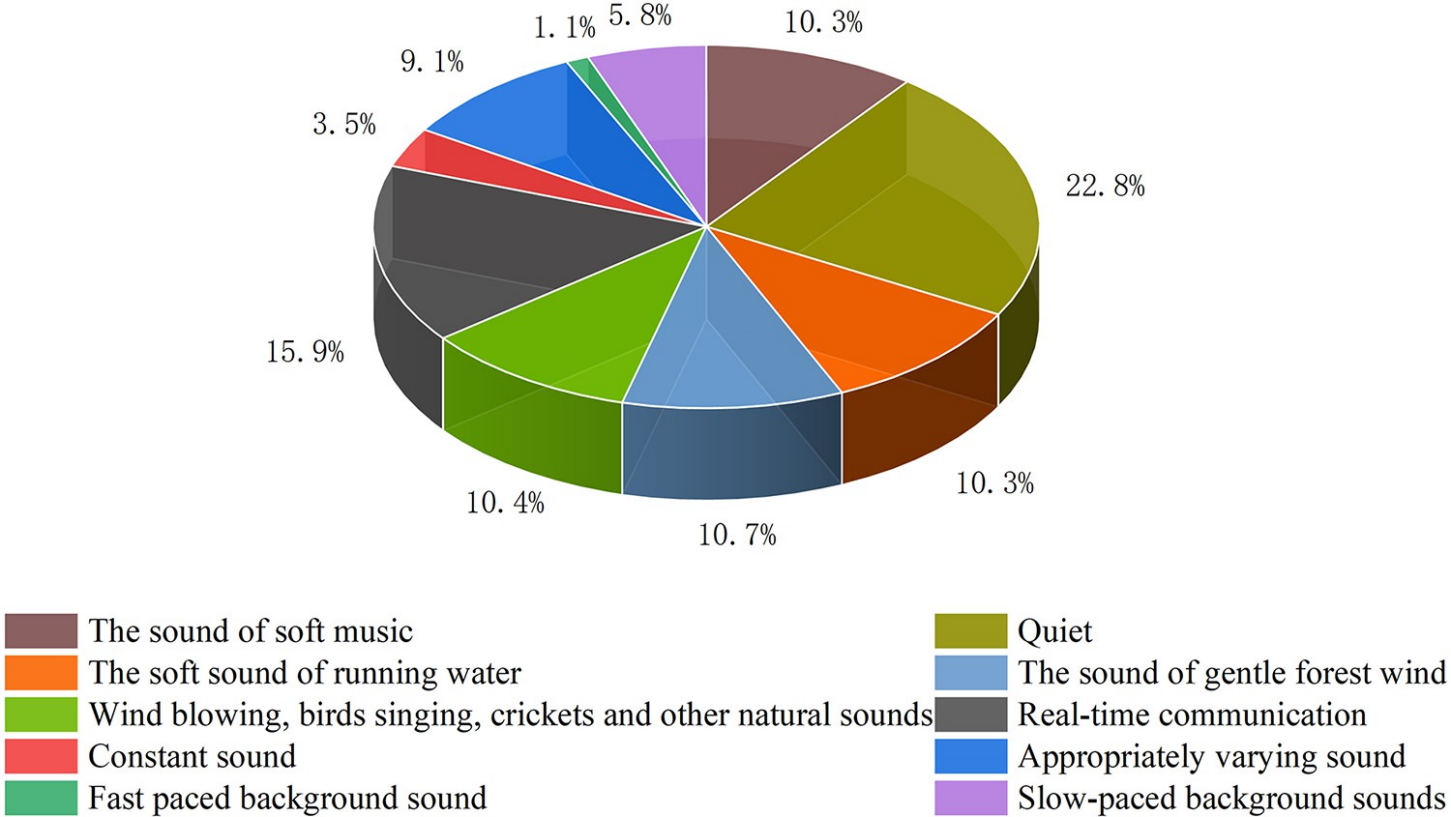
Votes for soundscape preferences.

During online classes at home, the soundscape can have a very important effect on the efficiency of learning. As shown in **Fig 4**, investigating the preferred soundscapes was helpful for understanding the preferences of students while receiving online classes at home. In terms of the overall classification, the subjects expressed preferences for natural and culturally relevant sounds, including the sounds of “a gentle forest wind” (10.7%), “wind blowing, birds singing, crickets, and other natural sounds” (10.4%), “the soft sound of running water” (10.3%), and “the sound of soft music” (10.3%). In terms of the overall number of votes, “quiet” soundscapes (22.8%) were the most popular, followed by respondents who wanted “real-time communication” (15.9%) in the online learning process in order to break the space restrictions in the same manner as an offline class. “Fast-paced background sound” (1.1%) was the least popular and only 1.1% preferred this soundscape during online classes. Fewer people preferred “constant sound” (3.5%) and “slow-paced background sounds” (5.8%) in the online class soundscape. Most preferred a quiet or natural and cultural soundscape, and they wanted to communicate actively to improve the efficiency of learning.

### 3.2. Learning efficiency

Learning efficiency is influenced by a complex interplay of various factors. To unravel this complexity, we focused on the impact of demographic characteristics and soundscape perceptions on learning efficiency among respondents engaged in home-based online classes.

#### 3.2.1. Effects of demographic characteristics

In addition to the effects of soundscapes on the efficiency of learning, differences in personal attributes can have important influences. Demographic factors comprising gender, age, region, and educational level are shown in **Table 6**.

**Table 6:** Average rating scores for educational attributes by demographic factor.

Average rating score Demographic factor		Concentration/Distraction	Interesting/Boring	Meaningful/Meaningless	Improved learning efficiency/Decreased learning efficiency	Favorable for communication/Unfavorable for communication
Age	≤ 17 years	0.25	0.00	–0.25	0.00	–0.50
	18–20 years	–0.13	–0.17	0.04	–0.39	–0.45
	21–25 years	–0.07	–0.14	0.14	–0.32	–0.46
	26–30 years	–0.62	–0.62	–0.54	–0.59	–0.43
	≥ 31 years	–0.28	–0.52	–0.57	–0.30	–0.42
Gender	Male	–0.24	–0.29	–0.11	–0.53	–0.47
	Female	0.01	–0.10	0.21	–0.12	–0.43
Region	North China	–0.08	–0.11	0.09	–0.27	–0.47
	South China	–0.19	–0.33	–0.04	–0.44	–0.43
Educational level	High school degree and below	–0.19	0.18	0.56	–0.07	–0.82
	College degree	0.32	–0.63	–0.59	0.15	–0.66
	Bachelor degree	–0.10	–0.20	0.05	–0.37	–0.41
	Master’s degree	–0.43	–0.22	–0.04	–0.51	–0.45
	PhD degree and above	–1.70	–0.50	–1.40	–1.30	–0.60

The ages of the respondents were categorized as ≤ 17, 18–20, 21–25, 26–30, and ≥ 31 years. In terms of “concentration/distraction,” the average rating score was lower for respondents aged 26–30 years and higher for those aged ≤ 17 years old. Those aged 26–30 and ≥ 31 years had lower average ratings for “interesting/boring,” and the ratings were higher for respondents aged ≤ 17 years. In terms of “meaningful/meaningless,” the average evaluation scores were lower for respondents aged 26–30 and ≥ 31 years, and higher for those aged 21–25 years, where the difference was 0.71. In terms of “improved learning efficiency/decreased learning efficiency,” respondents aged 26–30 years had lower average evaluation scores, and those aged ≤ 17 years had higher evaluation scores. In terms of “favorable for communication/unfavorable for communication,” the average rating scores were low for all age groups.

In terms of the effects of gender, the difference in “concentration/distraction” was 0.25, the difference in “meaningful/meaningless” was 0.32, and the difference in “improved learning efficiency/decreased learning efficiency” was 0.41. The evaluation scores were generally higher for women than men. In terms of region, there were no obvious differences between the evaluations given by respondents in North and South China, although the evaluations given by those in North China were generally higher.

Educational level saw doctoral and higher degree holders scoring lower in “centralization/distribution,” while junior college attendees rated higher, evidencing a significant disparity of 2.02. “Interesting/boring” scores were lesser among college and doctoral-plus degree students, with high school and below respondents rating it higher, marking the widest gap at 0.81. “Meaningful/meaningless” and “improved learning efficiency/decreased learning efficiency” followed similar patterns, with the most pronounced differences being 1.96 and 1.45, respectively, in favor of lower educational levels. However, “favorable for communication/unfavorable for communication” saw negligible variation across educational tiers.

#### 3.2.2. Soundscape and learning efficiency

**Fig 5:**
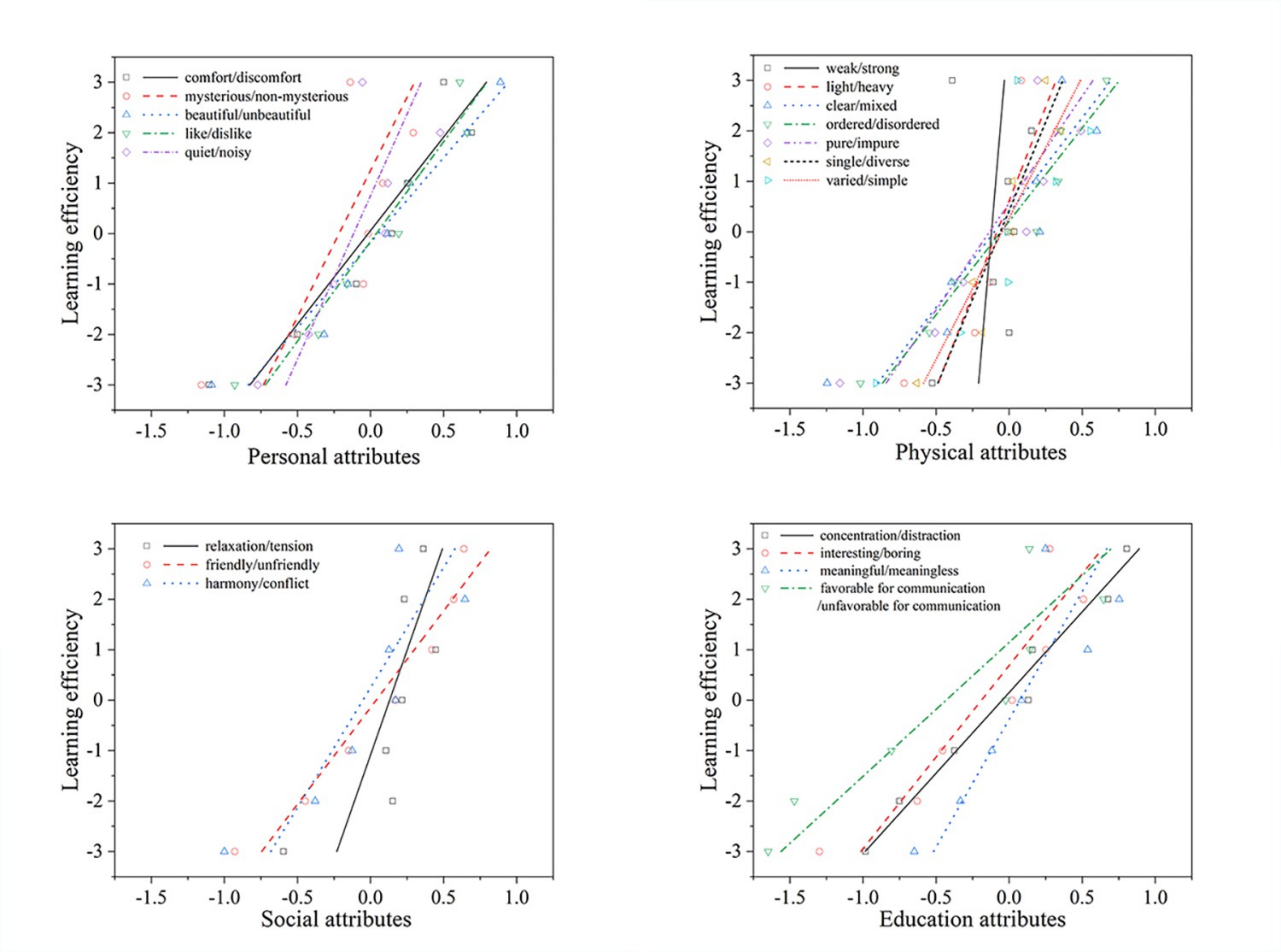
Relationships between four attributes of indoor soundscapes and the learning efficiency.

This subsection delves into the relationship between indoor soundscapes and learning efficiency during online classes, examining how various attributes—personal, physical, social, and educational—affect this dynamic, as shown in **Fig 5**.

Within personal attributes, the aspects of "comfortable/uncomfortable," "beautiful/not beautiful," and "like/dislike" exhibited significant correlations with learning efficiency, boasting R^2^ values of 0.888, 0.949, and 0.931, respectively. Meanwhile, "mysterious/not mysterious" and "quiet/noisy" held R^2^ values of 0.591 and 0.667, indicating a moderate impact. The data suggest that sounds perceived as comfortable and beautiful by students are conducive to enhanced learning efficiency.

For physical attributes, "light/heavy," "clear/mixed," "ordered/disordered," "pure/impure," and "single/diverse" all demonstrated strong correlations with learning efficiency, with R^2^ values of 0.761, 0.831, 0.941, 0.811, and 0.865, respectively. Conversely, "change/no change" showed a lower R^2^ value of 0.668, and "weak/strong" had a minimal influence on learning efficiency, indicated by an R^2^ value of only 0.066.

In the realm of social attributes, "friendly/unfriendly" and "harmony/conflict" were strongly correlated with learning efficiency, with R^2^ values of 0.947 and 0.754, respectively. However, "relaxation/tension" had a relatively minor impact, evidenced by an R^2^ value of 0.580.

Educational attributes, including "concentration/distraction," "interesting/boring," "meaningful/meaningless," and "favorable for communication/unfavorable for communication," all significantly influenced learning efficiency.

### 3.3. Semantic differential analysis

#### 3.3.1 Influencing factors of soundscapes subjective evaluation

Leveraging data garnered from the home-based online class survey, the study applied the correlation coefficient matrix across evaluation indices to determine their appropriateness for factor extraction. Varimax rotated principal component analysis via SPSS facilitated the derivation of orthogonal factors from the 20 pairs of parameters, pinpointing the principal factors influencing subjective soundscape evaluations, as shown in **Table 7**.

**Table 7:** Factor analysis based on all data obtained from indoor online class soundscape evaluations.

Semantic indicators	Factor			
	1 (17.115%)	2 (13.492%)	3 (10.189%)	4 (10.010%)
Clear/Mixed	0.703	0.199	0.250	0.147
Ordered/Disordered	0.680	0.150	0.117	0.226
Concentration/Distraction	0.640		0.133	0.324
Harmony/Conflict	0.639	0.303	0.149	
Friendly/Unfriendly	0.545	0.530	0.170	
Like/Dislike	0.501	0.479		0.156
Quiet/Noisy	0.463	0.148	0.409	0.209
Varied/Simple	–0.163	0.632	0.197	0.311
Relaxation/Tension	0.297	0.577	0.166	–0.232
Comfort/Discomfort	0.357	0.561		0.198
Mysterious/Not mysterious	0.174	0.529		0.168
Beautiful/Not beautiful	0.485	0.526		0.160
Weak/Strong		0.194	0.691	
Single/Diverse	0.214		0.663	
Light/Heavy	0.184	0.201	0.595	
Pure/Impure	0.303	0.171	0.487	0.331
Favorable for communication/unfavorable for communication	0.144			0.769
Improved learning efficiency/Decreased learning efficiency.	0.300	0.125		0.689
Interesting/Boring	0.268	0.357	0.190	0.421
Meaningful/Meaningless	0.149	0.376	0.297	0.385

Note

factor “1” refers to online course, factor “2” refers to relaxation, factor “3” refers to physical attributes, and factor “4” refers to personal learning.

KMO measure of sampling adequacy: 0.931, cumulative percentage: 50.806%; extraction method: principal component analysis; rotation method: varimax-rotation method; N = 951

The findings from the semantic differential analysis of indoor soundscapes during home-based online classes revealed significant insights into the factors that shape students’ auditory experiences. Factor 1, accounting for 17.115% of the variance, was closely linked to aspects of the online course itself, specifically clarity (clear/mixed), organization (ordered/disordered), and cohesiveness (harmony/conflict). Factor 2, explaining 13.492% of the variance, was associated with relaxation, encompassing interpersonal dynamics (friendly/unfriendly), emotional state (relaxation/tension), and overall comfort (comfortable/uncomfortable), along with aesthetic perceptions (mysterious/not mysterious and beautiful/not beautiful). Factor 3, representing 10.189% of the variance, pertained to physical attributes of sound, including intensity (weak/strong), variety (single/diverse), and weight (light/heavy). Factor 4, with 10.010% of the variance, focused on personal learning elements, highlighting communication (favorable for communication/unfavorable for communication) and the direct impact on learning efficiency (improved learning efficiency/decreased learning efficiency).

The predominant influence of the online course component (Factor 1) over other factors underscores the integral role of course design and delivery in shaping the soundscape perceptions and, by extension, the learning environment. Despite these insights, the combined factors accounted for just over half of the total variance (50.806%), a proportion somewhat lower than typically observed in soundscape evaluations. This discrepancy likely stems from the diversity in respondent locations, which introduces variations in acoustic environments and, consequently, in soundscape perceptions. Furthermore, the array of sound sources—differing not only in type and number but also in individual characteristics—adds another layer of complexity. Lastly, the inherent differences in how respondents perceive and interpret soundscapes suggest that personal background and auditory preferences significantly contribute to the subjective evaluation of soundscapes during online learning.

#### 3.3.2 Gender differences

To discern gender-based differences in soundscape perceptions during online classes, factor analysis was conducted separately for males and females across the collected data set. The outcomes, as detailed in **Tables 8** and **9**, reveal distinct patterns in how each gender relates to the auditory environment of home-based online learning.

**Table 8:** Factor analysis based on data provided by males in indoor online class soundscape evaluations.

Semantic indicators	Factor			
	1 (21.132%)	2 (11.577%)	3 (10.572%)	4 (8.391%)
Like/Dislike	0.693	0.227	0.160	
Friendly/Unfriendly	0.688		0.208	0.297
Mysterious/Not mysterious	0.673			
Comfort/Discomfort	0.665	0.189		0.156
Beautiful/Not beautiful	0.641	0.197		0.283
Harmony/Conflict	0.588	0.180	0.191	0.231
Relaxation/Tension	0.538		0.266	0.181
Clear/Mixed	0.523	0.227	0.267	0.372
Varied/Simple	0.428	0.394	0.183	–0.316
Quiet/Noisy	0.423	0.292	0.357	0.106
Favorable for communication/unfavorable for communication		0.806		0.151
Improved learning efficiency/Decreased learning efficiency.	0.165	0.786		0.194
Meaningful/Meaningless	0.324	0.416	0.296	0.104
Interesting/Boring	0.397	0.413	0.285	
Single/Diverse			0.728	0.187
Weak/Strong			0.672	
Pure/Impure	0.280	0.351	0.565	
Light/Heavy	0.337	0.189	0.428	–0.109
Concentration/Distraction	0.243	0.175	0.156	0.742
Ordered/Disordered	0.372	0.208		0.678

Note

factor “1” refers to relaxation, factor “2” refers to personal learning, factor “3” refers to physical attributes, and factor “4” refers to online course and personal learning.

KMO measure of sampling adequacy: 0.916; cumulative percentage: 51.672%; extraction method: principal component analysis; rotation method: varimax-rotation method; N = 551

**Table 9:** Factor analysis based on data provided by females in indoor online class soundscape evaluations.

Semantic indicators	Factor			
	1 (19.194%)	2 (11.040%)	3 (10.800%)	4 (10.065%)
Harmony/Conflict	0.721	0.146	0.189	
Like/Dislike	0.672	0.195		0.169
Clear/Mixed	0.667	0.250	0.339	
Friendly/Unfriendly	0.663		0.141	0.299
Beautiful/Not beautiful	0.610	0.220		0.311
Ordered/Disordered	0.602	0.296	0.293	
Relaxation/Tension	0.580	–0.405		0.345
Favorable for communication/unfavorable for communication		0.741		0.175
Improved learning efficiency/Decreased learning efficiency.	0.266	0.622		0.101
Concentration/Distraction	0.387	0.511	0.301	0.113
Light/Heavy	0.174		0.672	0.325
Weak/Strong			0.656	0.411
Single/Diverse	0.101		0.637	–0.172
Quiet/Noisy	0.413	0.249	0.489	
Pure/Impure	0.383	0.331	0.439	
Varied/Simple				0.734
Meaningful/Meaningless	0.182	0.238	0.270	0.471
Interesting/Boring	0.267	0.398		0.457
Comfort/Discomfort	0.432	0.243		0.448
Mysterious/Not mysterious	0.150	0.343		0.378

Note

factor “1” refers to relaxation, factor “2” refers to personal learning, factor “3” refers to physical attributes, and factor “4” refers to online course.

KMO measure of sampling adequacy: 0.888; cumulative percentage: 51.089%; extraction method: principal component analysis; rotation method: varimax-rotation method; N = 440.

For male participants (N = 511), the analysis elucidated that four factors accounted for 51.672% of the total variance. Notably, Factor 1, contributing 21.132% to the variance, was predominantly associated with relaxation, encompassing a range of sentiments from like/dislike to comfort levels and aesthetic judgments like beautiful/not beautiful. Factor 2 (11.577%) revolved around personal learning, highlighting the significance of communication and the direct impact on learning efficiency. Factor 3 (10.572%), related to physical properties, emphasized the diversity and intensity of sound. Factor 4 (8.391%) merged aspects of online course structure with personal learning, including attention focus and organizational aspects of sound.

For female participants (N = 440), the factors accounted for 51.098% of the variance. Factor 1 (19.194%) similarly prioritized relaxation, mirroring the male preference for aesthetics and comfort but also including aspects of ease and tranquility. Factor 2 (11.040%) also focused on personal learning but added the element of concentration to the mix. Factor 3 (10.800%) dealt with physical attributes, paralleling male concerns but placing emphasis on different aspects of sound intensity and diversity. Factor 4 (10.065%) related specifically to the online course environment, focusing on the variety and simplicity of sound.

Despite these nuanced differences, the overarching categories of relaxation, personal study, physical attributes, and online courses were consistent across genders, affirming relaxation as the most influential factor in soundscape evaluations for both males and females. However, the focus on specific semantic indicators varied, with males attending to a broader range of factors, while females exhibited selective sensitivity to fewer aspects.

#### 3.3.3 Regional differences

The investigation into regional differences between respondents from South and North China provides insightful distinctions in how indoor soundscapes during online classes are perceived and which aspects are deemed most influential on learning efficiency. **Tables 10** and **11** delineate the factor analysis outcomes based on the comprehensive data set for both regions.

**Table 10:** Factor analysis based on data provided by respondents from South China in indoor online class soundscape evaluations.

Semantic indicators	Factor			
	1 (23.955%)	2 (11.046%)	3 (10.947%)	4 (8.118%)
Friendly/Unfriendly	0.755			0.187
Beautiful/Not beautiful	0.722	0.167		0.174
Clear/Mixed	0.708	0.228	0.300	–0.151
Harmony/Conflict	0.699	0.201	0.244	
Relaxation/Tension	0.652	–0.280	0.135	0.290
Ordered/Disordered	0.626	0.326	0.108	
Comfort/Discomfort	0.607	0.206		0.294
Like/Dislike	0.589	0.320	0.159	0.224
Concentration/Distraction	0.517	0.364	0.180	
Mysterious/Not mysterious	0.428	0.104	0.108	0.218
Favorable for communication/unfavorable for communication	0.124	0.806		0.210
Improved learning efficiency/Decreased learning efficiency.	0.328	0.659		0.166
Interesting/Boring	0.398	0.429		0.294
Single/Diverse			0.746	
Pure/Impure	0.371	0.281	0.602	
Weak/Strong		–0.180	0.596	0.384
Light/Heavy	0.209		0.553	0.166
Quiet/Noisy	0.367	0.345	0.521	–0.152
Varied/Simple		0.239		0.786
Meaningful/Meaningless	0.347	0.232	0.251	0.529

Note

factor “1” refers to relaxation, factor “2” refers to personal learning, factor “3” refers to physical attributes, and factor “4” refers to online course.

KMO measure of sampling adequacy: 0.885; cumulative percentage: 54.066%; extraction method: principal component analysis; rotation method: varimax-rotation method; N = 389.

**Table 11:** Factor analysis based on data provided by respondents from North China in indoor online class soundscape evaluations.

Semantic indicators	Factor			
	1 (15.747%)	2 (12.958%)	3 (11.112%)	4 (9.987%)
Concentration/Distraction	0.709		0.330	0.114
Ordered/Disordered	0.684	0.124	0.228	0.163
Clear/Mixed	0.664	0.200	0.229	0.244
Harmony/Conflict	0.602	0.332		0.150
Like/Dislike	0.544	0.464	0.120	
Friendly/Unfriendly	0.518	0.509		0.270
Quiet/Noisy	0.408	0.228	0.262	0.371
Mysterious/Not mysterious		0.678	0.245	
Beautiful/Not beautiful	0.391	0.590	0.209	
Comfort/Discomfort	0.322	0.573	0.195	
Varied/Simple		0.568	0.171	0.316
Relaxation/Tension	0.294	0.454	–0.214	0.308
Favorable for communication/unfavorable for communication	0.101		0.712	0.102
Improved learning efficiency/Decreased learning efficiency.	0.257	0.103	0.695	
Pure/Impure	0.131	0.249	0.503	0.360
Interesting/Boring	0.247	0.276	0.460	0.237
Meaningful/Meaningless	0.190	0.218	0.455	0.263
Weak/Strong			0.125	0.723
Light/Heavy	0.227	0.140		0.629
Single/Diverse	0.231		0.137	0.525

Note

factor “1” refers to online course, factor “2” refers to relaxation, factor “3” refers to personal learning, and factor “4” refers to physical attributes.

KMO measure of sampling adequacy: 0.921; cumulative percentage: 49.804%; extraction method: principal component analysis; rotation method: varimax-rotation method; N = 562.

For the South China cohort (N = 389), the analysis highlighted that four factors accounted for 54.066% of the total variance. Specifically, Factor 1, which represented 23.955% of the variance, was primarily associated with relaxation, incorporating elements such as friendliness, aesthetic appreciation, ease, comfort, and preference. Factor 2 (11.046%) focused on personal learning aspects, emphasizing the importance of communication and its impact on learning efficiency. Factor 3 (10.947%) was connected to physical attributes of sound, highlighting diversity, purity, strength, and weight. Factor 4 (8.118%) pertained to the online course itself, including variety and focus.

Conversely, in North China (N = 563), Factor 1 was closely tied to the online course, comprising aspects of order, clarity, and harmony. Factor 2, emphasizing relaxation, included friendliness, mystery, beauty, and comfort. Factor 3 concentrated on personal learning, underscoring communication and the direct effects on learning efficiency. Lastly, Factor 4 addressed physical attributes, focusing on strength, weight, and diversity. Together, these factors explained 49.804% of the total variance, a slightly lower coverage than observed in the South.

This comparative analysis reveals "relaxation" as the predominant factor in the South, significantly influencing soundscape evaluations during home-based online classes. This contrasts with the North, where "online courses" and "relaxation" were of paramount importance but presented differently from the Southern preference.

The regional distinction underscores the necessity of considering geographical and cultural contexts in designing and delivering online education. It suggests that while certain elements like relaxation and course structure universally impact learning, the emphasis and prioritization of these factors can vary significantly across different regions. Tailoring educational content and the acoustic environment to regional preferences may enhance the online learning experience, potentially boosting engagement and efficiency for students across diverse locales.

## 4. Discussion

### 4.1 Soundscape evaluation

#### 4.1.1. Subjective evaluations of sound level and acoustic comfort

Historically, the evaluation of sound has relied heavily on physical sound indicators measured by sound level meters, such as average sound pressure levels or equivalent sound pressure levels, expressed in decibels (dB). These metrics form the foundation of many international regulations and guidelines. Yet, as the field of soundscape research has evolved, it has unveiled the nuanced relationship between sound pressure levels and subjective sound evaluations, noting that perceptions can significantly diverge based on differing research contexts. For example, Juhani Parmanen highlighted in 2007 that while A-weighted sound pressure level is a reasonable gauge of environmental sound annoyance, it does not precisely reflect subjective loudness [32]. Similarly, Meng Qi’s 2010 research demonstrated how environmental sound levels and subjective sound comfort are related, showing that comfort peaks at around 65dB before declining [33]. Jin Yumeng’s 2020 study further corroborated that lower sound pressure levels are associated with higher subjective sound comfort in outdoor environments [34]. Educational sound environments have also been scrutinized, revealing that high noise levels in classrooms can lead to teacher fatigue and irritability, as per Rabelo’s 2015 study [35], while FAM Dias’s 2019 research [36] found that classroom noise adversely affects students’ concentration, performance, and lecture clarity.

This study’s lack of correlation between subjective loudness, sound comfort evaluations, and average sound pressure levels suggests that the context of sound evaluation—particularly in home-based online learning environments where the teacher’s voice is predominant—differs significantly from that of public spaces or leisure environments. This context specificity confirms the complex interplay between sound perception and the educational setting, underscoring the need for more nuanced soundscape management strategies in online learning environments.

#### 4.1.2 Sound perceptions and preferences in home teaching

For sound perceptions and preferences, a large number of exploratory studies [37–39] based on public spaces have shown that people significantly prefer natural sounds (such as bird calls, wind, water, etc.) over human sounds (such as children’s shouting, surrounding speech, and footsteps) and other man-made sounds (such as mechanical noises). Regarding the indoor sound environment for educational purposes, research has found that students generally dislike sound sources mostly coming from outside the classroom [12]. Among the sound sources inside the classroom, students least like the noise from electromechanical equipment such as air conditioners and alarms, whereas the sound of teachers typing on keyboards is not only not repelled by students but also somewhat accepted. The study suggests that students are likely to accept sound sources related to education.

In this study, the sound of teachers giving online lectures was the most accepted by the participating students, followed by natural soundscapes with quiet characteristics such as wind and rain sounds. The least popular were the sounds of the television at home, parents talking, outdoor traffic noise, and notification sounds from communication software. This result encompasses the characteristics of sound perceptions and preferences both in outdoor public spaces and classroom environments. That is, students are more likely to accept sound sources related to education, and at the same time, they prefer to have quiet or natural and cultural atmospheres in the soundscape, which can not only help reduce fatigue but also improve learning efficiency.

### 4.2 Learning efficiency

Learning efficiency is crucial for online teaching; efficient learning can help students better absorb the course content. However, the average learning efficiency score was –0.341 according to the subjective questionnaire, which shows that the learning efficiency was somewhat reduced for respondents who received online classes at home. Further analysis of the impact of different soundscape attributes on learning efficiency reveals, the effects of personal, physical, social, and educational attributes of soundscapes on the learning efficiency are shown in **Table 12**, which demonstrates that “concentration/distraction” had the strongest correlation with the learning efficiency and “weak/strong” was closely related to the learning efficiency. This indicates that in the home learning environment, "concentration/dispersion" has a particularly significant impact on learning efficiency, while the intensity of sound is not a key factor affecting learning efficiency. Making the soundscape less diverse (“single”) could improve the learning efficiency most rapidly. This means that by reducing background noise and distracting sounds in the environment, creating a more unified and focused sound environment can effectively enhance learning efficiency. Making the soundscape more “favorable for communication” is the slowest way of improving the learning efficiency. Therefore, based on the research in this paper, the planning and design of soundscapes should be emphasized. If a more singular soundscape can be created during home online learning, making the environmental sound lighter, the soundscape quieter, and these soundscapes capable of inducing relaxation, it can quickly enhance learning efficiency.

**Table 12:** R^2^ and K values after fitting the four attributes with learning efficiency.

	Semantic indicators	R^2^	K
Personal attributes	Comfort/Discomfort	0.88776	3.29231
	Mysterious/Not mysterious	0.59129	3.43495
	Beautiful/Not beautiful	0.94862	3.19529
	Like/Dislike	0.93132	3.68262
	Quiet/Noisy	0.6673	4.32395
Physical attributes	Weak/Strong	0.06637	2.26267
	Light/Heavy	0.76114	5.65734
	Clear/Mixed	0.83109	3.12447
	Ordered/Disordered	0.94126	3.48553
	Pure/Impure	0.81147	3.43996
	Single/Diverse	0.8652	6.07276
	Varied/Simple	0.66821	3.73798
Social attributes	Relaxation/Tension	0.58036	4.82051
	Friendly/Unfriendly	0.94743	3.62944
	Harmony/Conflict	0.75376	3.5861
Educational attributes	Concentration/Distraction	0.97628	3.12356
	Interesting/Boring	0.87102	3.16389
	Meaningful/Meaningless	0.7609	3.85445
	Favorable for communication/unfavorable for communication	0.84782	2.25154

### 4.3 Semantic differential analysis

As indicated in **Table 13**, we can compare papers from different research directions with this paper. Kang et al.’s research primarily focused on urban public open spaces, with samples including students and the general public [15]. Liu et al.’s study involved forest spaces, classifying Chinese tourists’ preferences for single sound sources versus the overall sound environment in forest settings [40]. Yang et al. conducted research on ancient town spaces, based on tourists’ sound preferences, categorizing them into those favoring natural sounds and those favoring artificial sounds [41]. Li et al.’s research direction was campus spaces, selecting semantic indicators that cover the physical, social, and personal attributes of soundscapes to evaluate campus soundscapes [42]. Acun’s research focused on indoor soundscapes, particularly open learning area spaces, with samples being students within university campuses [8]. This paper further narrows down the scope to indoor spaces during home online classes, a relatively new yet inevitable scenario, somewhat filling the research gap with samples covering respondents from multiple provinces in China. In terms of research methods, this study and other papers mainly used the semantic differential method, but specific evaluation indicators and sample characteristics differ. For example, this paper pays special attention to the special learning environment of online classes at home, which might influence respondents’ perceptions and preferences towards soundscapes.

**Table 13:** Comparison of different soundscape evaluation factors.

Research area	Number of factors	Factors	Cumulative percentage explained variance
**Indoor soundscape during online class**	**4**	**Online course, Relaxation, Physical attributes, Personal learning**	**50.806%**
Urban open public space soundscape	4	Relaxation, Communication, Spatiality, Dynamics	53%
Forest soundscape	4	Leisure and entertainment, Spatial characteristics, Sound quality and dynamics, Environmental perception	61.054%
Ancient town soundscape	5	Size, Sound quality, Valence, Preference, Arousal	62.36%
Campus soundscape	3	Life, Communication, Spatiality	About 60%
Open study area soundscape	3	Sensation, Activity/Communication, Functionality	58.8%

In terms of research findings, according to Kang Jian et al.’s study in urban public open spaces in Sheffield, UK, the characteristics of soundscape semantic differential evaluation primarily include four leading factors: "relaxation, communication, space, and dynamism" [15]. When Liu Yiping et al. investigated the preference characteristics of Chinese tourists for forest soundscapes, they found that the four main factors affecting forest sound environment preferences were "leisure and recreation, spatial characteristics, sound timbre and dynamics, and environmental perception," indicating that when Chinese tourists are in forest soundscapes, they are more focused on leisure, recreation, and mental relaxation, with less attention to communication [4]. Yang Lingling et al., after using factor analysis to extract factors of tourists’ subjective evaluations of the soundscape in Lijiang Dayan Ancient Town, found five factors: "volume, timbre, valence, preference, and arousal," which have a certain hierarchy. The lower-level evaluations lean towards the physical properties of the sound itself, while the higher levels lean towards the psychological effects produced by the sound [5]. According to Li Zhuying et al.’s study on campus soundscapes, the three main factors affecting campus soundscape evaluations were "life, communication, and sense of space," showing the complex characteristics of campus soundscapes [42]. In terms of indoor soundscapes, Acun Volkan et al. studied the soundscape of open learning areas, extracting three factors: sensation, activity/communication, and function [8]. Comparing the leading factors and semantic indicators in this study with those, the dominant factors and coverage of semantic indicators of indoor soundscapes during home online classes lack the aspects related to activity and function, and the coverage rates of dominant factors and semantic indicators also differ. One reason is the different settings of semantic indicators; on the other hand, the respondent groups and the scenarios in which respondents are situated differ in this study. These different environments might lead to different soundscapes compositions and expectations towards soundscapes. When students are in the environment of online classes, they pay more attention to the content and experience of the courses.

## 5. Conclusion

The investigation into indoor soundscapes during home-based online classes has revealed the intricate and variable nature of auditory environments and their perception across diverse demographic segments. Drawing from our comprehensive analysis of soundscapes and related evaluations in such settings, several key conclusions emerge:

1. **Sound Source Preferences:** Respondents exhibited a pronounced preference for sounds emanating from nature and culture, alongside a general acceptance of lecture-related sounds during online classes. Notably, there was a strong inclination towards quiet, natural, and culturally rich soundscapes that also facilitate active communication, which in turn, was perceived to augment learning efficiency.
2. **Identification of Key Factors:** Utilizing SPSS for varimax rotated principal component analysis on the semantic differential analysis data, we delineated four principal factors impacting indoor soundscapes in home-based online classes: "online course," "relaxation," "physical attributes," and "personal learning." These findings underscore the importance of focusing on these dimensions when evaluating and enhancing home-based online learning environments.
3. **Impact on Learning Efficiency:** The diverse soundscapes encountered in home-based online classes were found to significantly influence learning, with 45.7% of respondents indicating a perceived reduction in learning efficiency attributable to the soundscape. It was observed that learners’ perceptions varied across age, gender, region, and educational level. Crucially, our findings suggest that a simpler, quieter soundscape, characterized by minimal ambient noise, fosters a more relaxing and conducive learning environment, thereby potentially elevating learning efficiency.

## Supporting information

S1 Data (XLSX)
